# Development of the Systemic Lupus Erythematosus Steroid Questionnaire (SSQ): a novel patient-reported outcome tool to assess the impact of oral steroid treatment

**DOI:** 10.1186/s12955-017-0609-9

**Published:** 2017-02-28

**Authors:** Susan D. Mathias, Pamela Berry, Jane De Vries, Anca Askanase, Katie Pascoe, Hilary H. Colwell, David J. Chang

**Affiliations:** 1 Health Outcomes Solutions, PO Box 2343, Winter Park, FL 32790 USA; 2 GSK, King of Prussia, PA USA; 3 GSK, London, UK; 4 Columbia University Medical Center, New York, NY USA; 5 GSK, Philadelphia, PA USA

**Keywords:** Patient-reported outcomes, Oral steroids, Systemic lupus erythematosus, Impact, Steroid burden

## Abstract

**Background:**

Oral glucocorticoids (steroids) are the mainstay of treatment for systemic lupus erythematosus (SLE), but their use is often associated with short- and long-term side effects. Following a literature review and discussions with patients with SLE, clinicians, and payers, a need was identified for a comprehensive SLE-specific tool that can be used to evaluate the side effects and benefits of steroids over time from a patient perspective. The objective of this study was to develop a patient-reported outcome (PRO) measure to assess general impact (baseline burden), benefits, side effects, and impacts associated with the use of oral steroids in patients with SLE.

**Methods:**

A qualitative research protocol was developed in which adults with SLE currently receiving or who had received steroids in the past year were recruited from six US rheumatology practices to participate in concept elicitation (CE) interviews. The SLE Steroid Questionnaire (SSQ) was developed based on CE interview results and clinical input. Cognitive debriefing interviews with a second group of patients with SLE evaluated the content, clarity, and relevance of the items. The SSQ was refined using patient feedback, clinician review, and a translatability assessment. The protocol received central independent review board approval.

**Results:**

Thirty-three patients (52% moderate disease severity; 58% currently receiving steroids, mean dose 8.7 mg/day) completed CE interviews. Patients reported benefits, side effects, and impacts from steroids. The refined SSQ contains 50 items assessing steroid dose/duration (4 items), general impact (baseline burden; 19 items), benefits (7 items), work/productivity (3 items), side effects (10 items), emotions (6 items), and overall satisfaction (1 item).

**Conclusion:**

The SSQ is a unique PRO, developed using robust scientific methodology in accordance with the Food and Drug Administration PRO Guidance. It was designed to comprehensively assess the patient experience with steroid therapy and better understand the benefits and burden of steroids for patients with SLE.

## Background

Oral glucocorticoids (steroids) are the mainstay of treatment for several auto-immune and inflammatory disorders, including systemic lupus erythematosus (SLE). While providing significant clinical benefits to patients, their use is associated with both short- and long-term side effects, which increase with dose and duration of use [1–5]. Long-term steroid use can lead to life-limiting adverse events (AEs) and have a negative impact on quality of life (QoL) [6].

Perspectives on the risks and benefits of steroids may vary between patients, physicians, and policymakers (“payers”). In particular, the patient perspective or experience is not necessarily well understood or documented. A recent study found that patients with SLE thought their physician needed a better understanding of the psychosocial difficulties and pressures they face and that these should be considered when making treatment decisions [7]. The impact of steroid use and the value of steroid-sparing has only been partially explored within clinical trials. Clinical trials provide a controlled environment in which to explore short-term benefits of steroid sparing; AEs are collected, but the interplay between efficacy, safety, and QoL has only been partially resolved, and the moderating effect of these negative outcomes on the holistic benefit to patients is often not captured. At a Scientific Advisory Board meeting (‘The impact of corticosteroids in SLE’; March 2014; sponsored by GSK) physicians and payers concluded that whilst many physicians see the importance of sparing/replacing steroids, others may view them as an effective treatment with manageable side effects. Payers may also be reluctant to pay a premium for new therapies that replace or reduce the dose of steroids in the absence of evidence to demonstrate what steroid-sparing will deliver in terms of reducing side effects, QoL benefits, and/or cost reductions (meeting minutes on file).

In the treatment of SLE, there is clinical consensus that the dose of steroids should be kept as low as possible or eliminated [7, 8]. Understanding the risks and benefits of steroid treatment, dependent upon disease, dose and longevity of use, would inform the debate about their value and how they should be best utilized in treatment. Steroid use needs to be appropriately managed by clinicians and patients, and should include careful consideration of the appropriateness of other available treatment options, the impact of steroid induced AEs, and any follow-on healthcare costs. Furthermore, assessing the impact and benefit of primary SLE treatments can be challenging unless the effects of steroids are fully taken into account, due to the potentially confounding effect of steroid therapy on efficacy measures. Only when steroid prescribing is informed by robust evidence, can the true value of sparing steroids in long-term clinical practice be estimated.

A literature and background review, carried out to identify potential patient-reported outcome (PRO) tools that assess the impact of steroids in patients with SLE, failed to identify any appropriate, fit for purpose measures. The search identified 113 citations from ClinicalTrials.gov, PubMed, EMBASE, PROQOLID, and conference abstracts. Only one tool was identified; it was initially developed for use in patients who receive oral steroids for the treatment of immune thrombocytopenic purpura, and measures the frequency and level of distress of 33 symptoms patients experienced during the past 4 weeks [9]. It was deemed unsuitable due to its focus on symptoms alone, and the recall period was too long to address frequent fluctuations associated with symptoms and steroid use in patients with SLE. Due to frequent changes in SLE symptoms and response to treatment, it is essential that a PRO measure is sensitive to change over time [10].

Following the background review and discussions with patients, clinicians, and payers, a need was identified for a comprehensive SLE-specific tool that can be used to evaluate the side effects and benefits of steroids over time.

In 2009, the Food and Drug Administration (FDA) released a guidance document for the development of PROs that could be used in medical product development. The guidance recommends that an iterative development process should be undertaken to develop a content-valid measure with adequate measurement properties [11]. The guidelines recommend obtaining significant patient input to identify important concepts, developing a conceptual framework, and conducting cognitive interviewing of patients to assess the content, clarity, and relevance of the draft PRO. It is important that features such as the recall period, response options, question format, and translatability are considered.

The objective of this study was to develop a PRO measure, the SLE Steroid Questionnaire (SSQ), which can be used to assess general impact (baseline burden), benefits, side effects, and impacts associated with oral steroid use over time in patients with SLE.

## Methods

### Study population

Patients with a clinical diagnosis of SLE according to the American College of Rheumatology classification criteria [12] were recruited from six US rheumatology practices (Mid-Atlantic: Arlington, VA; Titusville, NJ; South: Gainesville, GA; Fort Lauderdale, FL; West Coast: Pacifica, CA; and Midwest: Lansing, MI). Patients were screened and selected with the aim of enrolling a diverse and representative sample, including African Americans, patients with a wide range of age, time since diagnosis and organ involvement, and some patients in paid employment. All patients were US residents between 18 and 75 years of age. Potential participants were approached either at the clinic or by telephone and invited to participate in the study by the study site personnel. The study participants contributed to the development of several PRO measures; 33 of the 41 patients comprising the full development sample were currently receiving steroids or had received steroids in the past year, and were therefore included in the development of the SSQ. Development of the other PRO measures will be reported elsewhere [13]. All patients provided written, informed consent upon enrollment in the study. Study sites and participants were remunerated fair market value for their participation.

### Development of the SSQ

**Fig. 1:**
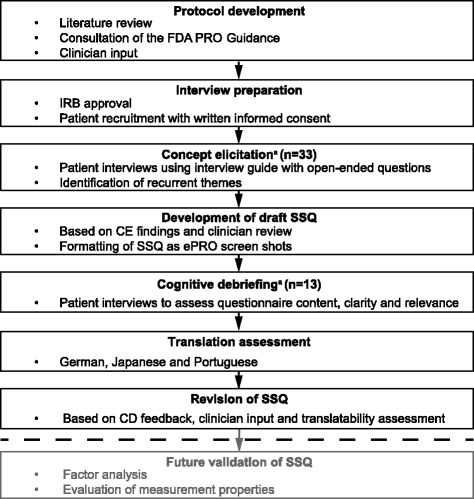
Development process of the SSQ. ^a^No patients took part in both the CE and CD interviews. CD, cognitive debriefing; CE, concept elicitation; ePRO, electronic patient-reported outcome; FDA, Food and Drug Administration; IRB, independent review board; PRO, patient-reported outcome; SSQ SLE Steroid Questionnaire

Figure 1 summarizes the scientifically rigorous development process used to develop the SSQ. The study protocol was developed and reviewed, then approved by an independent review board, the Copernicus Group. All sites received training on the conduct of the study and were required to complete a clinical case report form for each enrolled patient.

Patients with SLE participated in face-to-face concept elicitation (CE) interviews conducted by interviewers trained in PRO development. Demographic information was collected using a background questionnaire. Using a semi-structured interview guide, patients were asked open-ended questions about their steroid experience, including duration of steroid use and the benefits, side effects, and impacts of steroid use. While patients were questioned on the side effects of steroids, safety of other treatments was not formally assessed. Based on the results of these interviews and input from clinicians, the draft SSQ was developed.

To evaluate the content, clarity and relevance of the draft SSQ, face-to-face cognitive debriefing (CD) semi-structured interviews were conducted with a second sample of patients with SLE, recruited from four of the six US rheumatology practices. Prior to the face-to-face CD interview, patients completed a draft paper version of the SSQ formatted to look like screen shots, as the questionnaire will ultimately be administered using an electronic format (ePRO). In parallel with the CD interviews, a translatability assessment of the draft SSQ was performed to ensure easy translation into three representative languages: German, Japanese, and Portuguese.

The SSQ was refined based on the CD interview results, clinician input, and the translatability assessment results.

### Data analysis

All interviews were recorded and transcribed for analysis purposes. All data were held in accordance with local, state, and federal laws regarding confidentiality. Patient identifiable information was not included in any interview recordings, transcripts nor analyses, to ensure patient confidentiality.

Analyses were conducted using the full sample; however, due to the length of the interviews, not all questions in the CE and CD interviews were asked of all patients.

Data were analyzed using the qualitative data analysis software, MAXQDA (Verbi GmbH, Berlin, Germany). Two separate coding dictionaries were developed for analysis of the CE and CD interview transcripts. To ensure consistency across coders, descriptions and examples for each code were included. Each transcript was coded by one coder and then reviewed, summarized, and analyzed by a second coder.

## Results

### CE interviews

In June and July 2014, 41 patients with SLE completed CE interviews. Of these, 33 patients had a clinical record of steroid use in the previous year and completed interview questions about their experiences with steroids. The majority were female (97%), 50% were Caucasian, and 31% were African American; 19 patients were currently receiving steroids, with a mean (standard deviation) dose of 8.7 (5.35) mg/day (*n* = 18). The majority (82%) of patients were currently receiving concomitant medication, of which hydroxychloroquine was the most common (67%). Mean time since diagnosis was approximately 88 months (*n* = 17). Rheumatologists reported that approximately half (*n* = 17) of patients had moderate SLE and one patient had severe SLE. In the majority of cases, patients reported that it was the decision of the rheumatologist to initiate steroids (82%) and three patients (18%) stated that they made the decision jointly with their rheumatologist. The most common reason for initiating steroids was to treat an SLE flare (Table 1).

**Table 1:** Demographics and baseline clinical characteristics

	CE interviews^a^ (*N* = 33)
Gender, % (n)	
Male	3 (1)
Female	97 (32)
Mean age (SD), years	47.0 (11.13)
Age range, years	24–71
Ethnicity, % (n/N)	
Caucasian	50 (16/32)
African American	31 (10/32)
Asian	3 (1/32)
Latino/Hispanic	13 (4/32)
Other	3 (1/32)
SLE severity during 6 months prior to baseline, % (n)	
Mild	45 (15)
Moderate	52 (17)
Severe	3 (1)
Mean SELENA-SLEDAI (SD) (*n* = 30)	6.8 (3.36)
Mean SLICC (SD) (*n* = 18)	5.8 (1.54)
Current SLE treatment, %^b^ (n)	
Hydroxychloroquine	67 (22)
Belimumab	18 (6)
NSAIDs	15 (5)
Methotrexate	12 (4)
Currently receiving steroids, % (n)	58 (19)
Average steroid dose of current users (*n* = 18), mg/day	8.7 ± 5.35 (range: 4–20)
Mean (min, max) duration of steroid use (*n* = 23), months	41.7 (0.2, 144)
Reasons for initiating steroid use, % (n/N)	
To treat an SLE flare	26 (5/19)
To control swelling (hands or other body parts)	16 (3/19)
To treat joint pain and stiffness	16 (3/19)
To treat autoimmune thrombocytopenia/low platelet count	16 (3/19)

*CE* concept elicitation, *N* number of patients with data available, *NSAIDs* non-steroidal anti-inflammatory drugs, *SD* standard deviation, *SELENA-SLEDAI* Safety of Estrogens in Lupus Erythematosus National Assessment-Systemic Lupus Erythematosus Disease Activity Index, *SLE* systemic lupus erythematosus, *SLICC* systemic lupus international collaborating clinics

^a^Patients who answered questions regarding their steroid experience; where n does not equal 33, data were missing

^b^13 patients were receiving more than one treatment

The concepts mentioned with high frequency during the CE interviews are shown in Table 2. The most commonly reported side effects (≥10% of patients) were weight gain (67%), swelling/moon face (36%), mood swings/feelings of rage (21%), and difficulty sleeping (12%). Of patients currently taking steroids, 53% reported that they were not currently experiencing any side effects.

**Table 2:** Concepts discussed during CE interviews

	% (n/N) of patients (*n* = 33)^a^
Before receiving steroids	
Potential side effects discussed	71 (12/17)
Had expectations of the side effects	59 (10/17)
Perceived advantages of taking steroids	
It would be effective	31 (5/16)
Would have more energy	25 (4/16)
Would have less joint pain	25 (4/16)
Perceived disadvantages of taking steroids	
Weight gain	56 (9/16)
Potential long-term side effects eg, kidney problems and bone loss	38 (6/16)
After receiving steroids: side effects	
Expectations about side effects did not match experience^b^	50 (8/16)
Most commonly reported side effects (reported by ≥10% of patients)	
Weight gain	67 (22/33)
Swelling/moon face	36 (12/33)
Mood swings/feelings of rage	21 (7/33)
Difficulty sleeping	12 (4/33)
Most bothersome side effects	
Weight gain	64 (14/22)
Sleeplessness	14 (3/22)
After receiving steroids: dosage	
Have received a higher dose than they typically/currently take	85 (22/26)
Have received a lower dose than they typically/currently take	38 (10/26)
Stopped or changed dose without consulting physician	30 (8/27)
Satisfaction with steroid treatment	
Overall satisfied with steroids	75 (12/16)
Would be happy if treatment regimen did not include steroids	67 (12/18)

*CE* concept elicitation, *N* number of patients asked

^a^Patients who answered questions regarding their steroid experience; where n does not equal 33, data were missing/concepts were not discussed

^b^Most patients reported side effects were worse than expected

When asked how taking steroids made them feel (*n* = 19), in general, patients reported symptom improvement and increased energy. For example, one patient stated, “*I just felt like, you know like how you would feel on a really good day, you know you had the best night’s sleep and everything is going right, you’re having a great day and you have all the energy you need, the sun is shining. That’s what it was like*.” However, the negative impacts of steroids were also described, for example, “*The prednisone really helped the symptoms, but it made me feel sicker. Like you know the flares went away, but then I was overweight and bloated and you know my joints were swollen from the water. So I felt sicker even though I wasn’t having like you know massive flares.”*

When asked to put into one sentence their feelings about steroids (*n* = 16), many patients reported mixed feelings; steroids improved their SLE symptoms, but at the cost of experiencing side effects. For example, patients responded, “*I think they’re a two-edged sword because on one hand they help you and on the other hand they hurt you*” and “*I have a love/hate relationship with them.*” Overall, 75% of patients were satisfied with steroid treatment, while 67% of patients would be happy if they no longer needed to take steroids.

### Development of the SSQ

Based on analysis of the CE transcripts, a draft version of the SSQ was developed. Two rheumatologists then made minor revisions to the SSQ. For example, for consistency, items about side effects and benefits were revised to use the same response options. Additionally, a new item was added to assess the highest dose of steroids ever received and another item was added to enquire about feeling nervous or anxious.

The resulting draft questionnaire contained 51 items, which assessed steroid dose and duration (4 items), impact of steroids in general (18 items), benefits of steroids (8 items), work/productivity (3 items), side effects (10 items), emotions (7 items), and overall satisfaction (1 item).

### CD interviews and questionnaire refinement

To assess the content and clarity of the draft questionnaire, 13 patients with mild to moderate SLE completed CD interviews; 7/13 were currently treated with steroids and the other 6/13 had taken steroids within the past year. Interviews focused on questions that could have been challenging. For example, the question, did you experience reduced or less muscle pain or achiness?’ was debriefed, as it was thought that the term ‘achiness’ may have been difficult to understand.

All patients asked (*n* = 12) were able to accurately paraphrase the instructions on the SSQ and none (*n* = 10) had any suggestions for revisions.

The first four items in the SSQ ask about steroid dose and duration of treatment. Although all patients asked (*n* = 9) felt that it was easy to remember their current dose, 50% (5/10) of patients asked thought it was difficult to remember their highest dose.

Eighteen items ask patients about their general experience of steroids, including the benefits and side effects of steroids and the impacts of steroids on their daily life 75% of patients reported that it was easy to think about this. Fourteen items refer to specific benefits and side effects of taking steroids. When asked how easy or difficult it was to know if they were experiencing a side effect or benefit due to taking steroids or something else, 70% (*n* = 7/10) thought it was easy for most questions. Three patients thought that for more than one question it was difficult to know if the side effect was due to steroids; these included questions assessing energy level, mood, memory, daily activities, leisure activities, and desire to be intimate. For example, one patient responded: *“But, you know, when it comes to like my energy level I don’t know if that’s the steroids or the Lupus. The memory, I don’t know if that’s really with the two of them, or one of them, or…you know, some of them I don’t really know if it’s the steroids, the Lupus, or another medication that I might be on.”* Of patients who reported swelling or ‘moon face’, mood swings or feelings of rage, and weight gain, the majority (≥75%) reported that these occurred while on higher doses of steroids than while on their current dose. Some patients with mild SLE felt that some items were not relevant to them because they had not experienced them. For example, two patients reported having no difficulty sleeping and one patient reported not experiencing an increased appetite, feeling nervous/agitated, having a loss of memory, or being unable to concentrate.

When asked what time frame they considered when answering the questions on side effects, 71% of patients (*n* = 5/7) thought about the past 7 days, while 29% (*n* = 2/7) thought about the time since they first started taking steroids or in general. These results supported using a 7-day recall period for questions regarding steroid benefits and side effects in the second draft of the questionnaire. This time frame was also selected as it was considered to be short enough for patients to accurately recall their experiences, but long enough to ensure that a representative picture would be captured.

Seven items covered the range of emotions experienced in the last 7 days; most patients (*n* = 5/8) did not think there were any missing items. One patient suggested adding an item on feeling helpless or incapacitated, one suggested an item about feeling anxious, and one suggested an item about feeling stressed. As these items were suggested by a small number of patients, they were not added to the questionnaire.

Overall, respondent feedback was that the draft SSQ was comprehensive, clear, and relevant.

Based on the CD interviews, minor modifications were made to the SSQ to improve the clarity, including adding examples of side effects to the questions regarding short- and long-term side effects.

Minor changes were also made to the SSQ based on the translatability assessment. For example, the wording of the instructions “You may have been taking steroids for a short period of time or on and off for many years” was modified to “You may have been taking steroids for a short period of time, a long period of time, or you may have started and stopped steroids many times”, as the term “on and off” is difficult to translate into Japanese and German.

The refined SSQ contains 50 items; sample questions are shown in Table 3. Questions regarding the general impact of steroids do not specify a recall period and typically provide five response options, for example, much better, somewhat better, the same, somewhat worse, much worse. The majority of questions regarding the benefits and side effects of steroids use an 11-point numeric rating scale ranging from 0 (absent/did not have) to 10 (worst imaginable).

**Table 3:** Concepts included in the refined SSQ and sample questions

Concept (total number of items)	Sample questions and response options											
Steroid dose/duration (4)	What dose of steroids are you currently taking for lupus?	______mg/day OR ______mg ________(insert frequency, such as every day) OR _______ mg as needed OR □ I am not sure of my current dose										
General impact of steroids (baseline burden; 19)	In general, since you began taking steroids:											
	Do you feel that steroids have made your lupus symptoms…?	a) Much better b) Somewhat better c) The same d) Somewhat worse e) Much worse										
	How much of the time have you worried about short-term side effects of steroids (weight gain, mood changes, etc.)?	a) None of the time b) A little bit of the time c) Some of the time d) Most of the time e) All of the time										
Benefits of steroids (7)	While taking steroids for lupus in the past 7 days, to what degree did you experience joint pain?	0 Absent/Did not have	1	2	3	4	5	6	7	8	9	10 Worst imaginable
Work/productivity (3)	While taking steroids for lupus in the past 7 days, to what degree did you experience improved productivity at work?	a) Not at all b) A little c) Somewhat d) Quite a bit e) Very much										
Side effects (10)	While taking steroids for lupus in the past 7 days, to what degree did you experience increased appetite?	0 Absent/Did not have	1	2	3	4	5	6	7	8	9	10 Worst imaginable
Emotions (6)	While taking steroids for lupus in the past 7 days, how much of the time did you feel aggressive?	a) None of the time b) A little bit of the time c) Some of the time d) Most of the time e) All of the time										
	While taking steroids for lupus in the past 7 days, how much of the time did you feel self-confident?	a) None of the time b) A little bit of the time c) Some of the time d) Most of the time e) All of the time										
Overall satisfaction (1)	Overall, how satisfied have you been with how well steroids control your lupus symptoms?	a) Very satisfied b) Satisfied c) Neither satisfied nor dissatisfied d) Dissatisfied e) Very dissatisfied										

*SSQ* SLE Steroid Questionnaire

## Discussion

To our knowledge, this is the first comprehensive PRO measure to assess patient experience with oral steroids in SLE developed in accordance with the FDA PRO Guidance [11]. Patient and clinician input were obtained in all stages of the iterative development of the SSQ. This is an important criterion, as it ensures PROs are comprehensive and that they capture concepts that patients feel are most relevant.

The CE interviews captured the patients’ experience with steroid treatment and revealed the mixed attitudes patients have towards steroids, as they reported both positive and negative impacts of steroid therapy. The results of these interviews, together with input from rheumatologists, were used to develop the draft SSQ. Subsequently, patient CD interviews evaluated the clarity, comprehensiveness and relevance, and a translatability assessment was carried out in parallel. Minor modifications were made to the SSQ based on CD interview results, clinician input, and translatability results.

The refined SSQ includes 50 items that assess steroid dose/duration (4 items), impact of steroids in general (19 items), benefits of steroids (7 items), work/productivity (3 items), side effects (10 items), emotions (6 items), and overall satisfaction (1 item). Questions regarding the benefits and side effects of steroids use a 7-day recall period, which was determined through patient feedback and supported by clinicians, and enables changes in responses to be captured accurately. The sensitivity of the SSQ to time-dependent fluctuations is particularly important in SLE, a disease characterized by flares (periods of increased symptoms) followed by periods of quiescent disease or lower disease activity that might be accompanied by variations in the steroid dose. At 50 items, the SSQ may be viewed as being too lengthy for clinical use. However, it is important to note that the first 19 items assessing general experience with steroids are only asked once at baseline, with only the remaining 31 items asked on an ongoing basis.

The SSQ is the first tool designed to evaluate the overall steroid experience in SLE. It captures both general experiences (baseline burden), and the patient’s recent experience of steroids. Importantly, it goes beyond assessing the negative impacts associated with steroid therapy (side effects) by also investigating the positive impacts, such as improvement in SLE symptoms. In some cases, patients reported that it was difficult to distinguish side effects caused by steroids from symptoms of the disease, or other treatment side effects; this may limit the ability of the SSQ to accurately attribute burden to steroid use. Longitudinal use and analysis of individual changes to the SSQ over time may help to better differentiate between the effects of steroids themselves, and other factors.

In addition to assessing the benefits, side effects and other patient impacts of steroids, it is important to take into account the wider economic consequences of steroid therapy. The severe side effects of steroids can generate an additional economic burden; for example, a recent study estimated the annual cost of steroid-related AEs (fracture, cataract, diabetes, peptic ulcer, stroke, myocardial infarction, and non-Hodgkin’s lymphoma) to be £165/year per steroid-treated patient (2007 GB pounds, equivalent to $344 [inflated to 2009 US$]) [14, 15]. Reducing the dose of steroids reduces the incidence of steroid-related AEs and therefore the economic burden of the therapy [14].

The 33 patients who completed the SSQ CE interviews had a wide range of steroid exposure (less than 1 month to 144 months) and captured the experience of patients who use steroids acutely and those who use them chronically. In addition, the sample captured the diversity of patients with SLE as it included both Caucasian and African American patients, as well as patients with a range of disease severity. Of the 33 patients enrolled in the CE interviews, only one was male. Although the incidence of SLE is much lower among men than women, [16] the inclusion of more men in the study population would have been preferable. Patients participating in the CD interviews were all of moderate or mild severity. Patients with mild SLE felt that some items were not relevant to them because they had not experienced the benefit or side effect, thus, some items (eg, having difficulty sleeping) of the SSQ may only be relevant to patients with moderate or severe SLE. Another potential limitation of this study was the length of the CD interviews. Not all questions could be asked of all patients; only a small number of patients reviewed each item, though interviews focused on items that were more challenging in terms of patient understanding. Further studies will be required to fully evaluate the SSQ in terms of its validity, reliability, sensitivity, and responsiveness to change over time (including a preliminary determination of a responder definition) before it can be used with confidence in clinical studies and real-world clinical practice. Traditional psychometric testing and exploratory factor analysis, should be conducted in order that both item and scale scores can be used in the analysis of SSQ data.

Once adequate measurement properties of the SSQ are demonstrated, the SSQ may have many uses in both clinical studies and clinical practice. The SSQ can be utilized in both cross-sectional and longitudinal studies. For example, within clinical studies the SSQ may assess the impact of steroids on specific SLE symptoms and impacts and to assess the steroid-sparing effect of a therapy. In the clinical practice setting, the baseline data provided by the SSQ could be used by physicians to assess the current burden of steroids on a patient. The SSQ will also help physicians better understand patients’ desires and concerns regarding steroid therapy, improve the physicians’ understanding of the impact of steroids, and help guide physician decisions regarding altering a patient’s steroid therapy. Although the SSQ was developed for use by patients with SLE, with modification and further validation, it could also be applicable to other patient populations in which steroid use is common, such as severe asthma or giant cell arteritis.

## Conclusions

In summary, the SSQ is a unique PRO measure designed to comprehensively assess the benefits, side effects, and other impacts of steroid treatment for patients. Following confirmation of its measurement properties, the SSQ may prove a useful tool in both clinical studies and clinical practice, to assess benefits and burdens associated with steroid use, and the steroid-sparing effect of therapies in SLE, with the potential for adaptation and use in other disease areas.

## Abbreviations

AE: Adverse event
CD: Cognitive debriefing
CE: Concept elicitation
ePRO: Electronic patient-reported outcome
FDA: Food and Drug Administration
NSAIDs: Non-steroidal anti-inflammatory drugs
PRO: Patient-reported outcome
QoL: Quality of life
SD: Standard deviation
SELENA-SLEDAI: Safety of estrogens in lupus erythematosus national assessment-systemic lupus erythematosus disease activity index
SLE: Systemic lupus erythematosus
SLICC: Systemic lupus international collaborating clinics
SSQ: SLE steroid questionnaire
